# Supplementary material for: Benchmarking the influence of pre-training on explanation performance in MR image classification
Source: Front Artif Intell. 2024 Feb 26;7:1330919. doi: 10.3389/frai.2024.1330919 (PMC10925627; doi:10.3389/frai.2024.1330919)
Supplement: Supplementary file 1 [file Data_Sheet_1.pdf]

Supplementary Material

1 HUMAN CONNECTOME PROJECT

Data were provided in part by the Human Connectome Project (HCP), WU-Minn Consortium (Principal Investigators: David Van Essen and Kamil Ugurbil; 1U54MH091657) funded by the 16 NIH Institutes and Centers that support the NIH Blueprint for Neuroscience Research; and by the McDonnell Center for Systems Neuroscience at Washington University. For more information, see the datasheet in section 10.

2 CODE AND DATA ACCESS

The code generating the lesion maps and performing the model training as well as running the explanation methods is available on Github: `https://github.com/Marta54/Pretrain_XAI_gt` . Please, find the generated lesion data under this link: https://www.doi.org/10.17605/OSF.IO/XNWAJ The lesions are generated using the function *create_lesion()* from the *Finetuning_on_2500_images.ipynb* notebook.

3 STEPS TO CREATE LESIONS.

Figure S1 displays the different steps, from the noise image to the final lesion needed to create the lesions to be added to the MRI background. These steps are thoroughly described in section 2 of the main body of this paper.

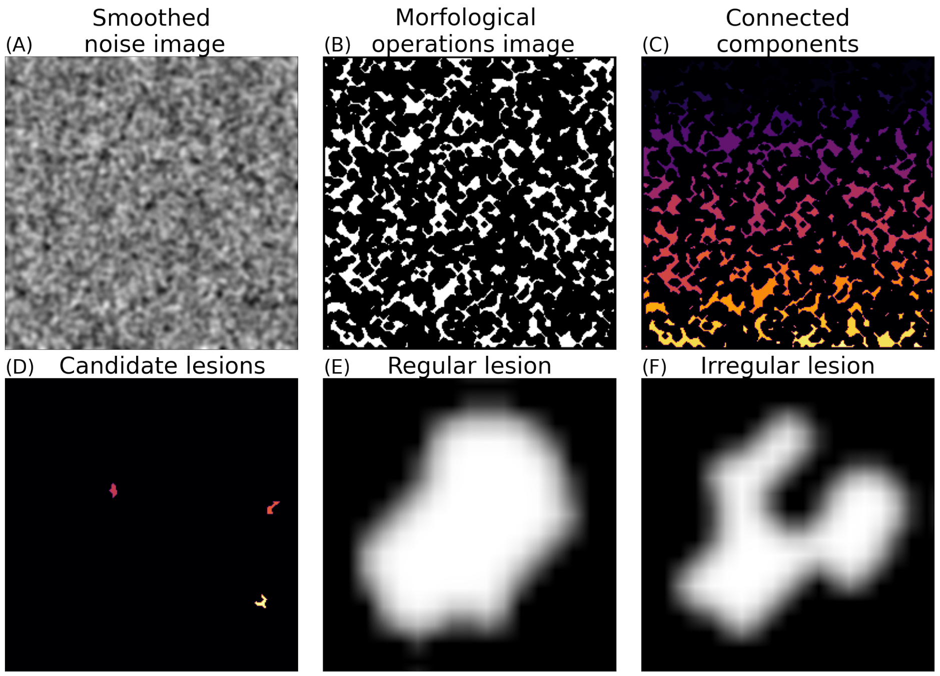

Figure S1. Steps to create lesions. A) A Gaussian smoothing filter with radius 2 pixels is applied to a 256×256 pixel white noise image. B) Erosion and Opening are applied to the binary version of (A). C) Connected components obtained from (B). D) Candidate lesions in (C) with size of 49 pixels. E) Regular lesion obtained from these steps. F) Irregular lesion obtained with the same method.

4 VGG-16 ARCHITECTURE

Figure S2 shows the different layers that build VGG-16 architecture. The purple layers represent convolutional layers, the yellow layers represent max-pooling layers, and the red layers represent fully-connected layers. In this architecture, max-pooling layers separate the NN into blocks of convolutional layers. These are the blocks used to identify five different degrees of fine-tuning.

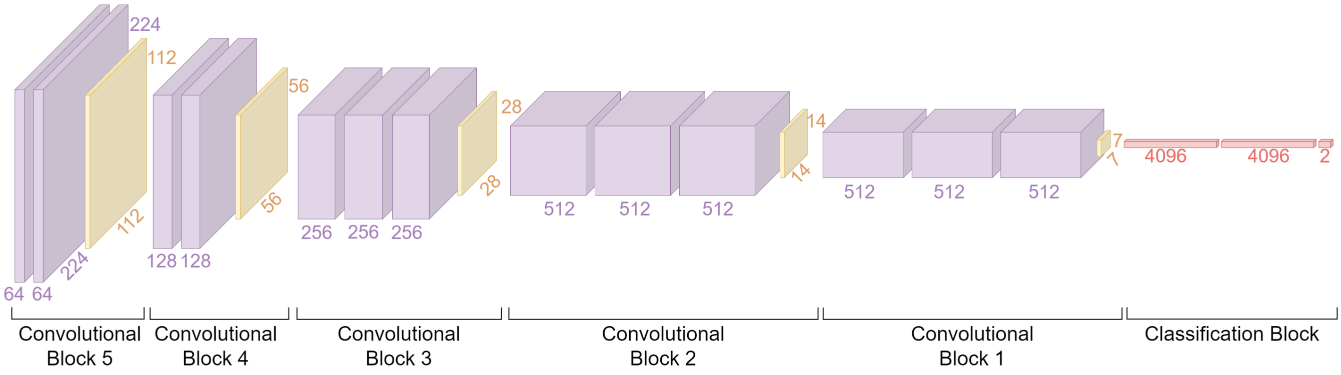

Figure S2. Scheme representing VGG-16 architecture and the blocks used to fine-tune the models. The purple layers represent convolutional layers, the yellow layers represent max-pooling layers, and the red layers represent fully-connected layers. The blocks mentioned below correspond to the divisions used to fine-tune the models.

To fine-tune the pre-trained model we used the holdout and validation datasets from the HCP. The data used to fine-tune the model consists of 2500 slices from the holdout dataset. This was done because the training dataset from the HCP had been already used to pre-train the model, which could create dependencies if the same data was used as the background of the fine-tuning dataset. Another 2500 slices from the same holdout dataset, were used to obtain the quantitative explanation performance. Lastly, 2500 slices from the validation dataset were used to validate the fine-tuning.

5 MODEL TRAINING

To train and fine-tune the models, we used an internal cluster equipped with GPUs of the type NVIDIA GeForce GTX 1080 Ti.

6 BASELINES OF XAI METHODS

Figure S3 displays the heat maps obtained by applying the XAI methods to a model randomly initialised and untrained. Here we can see that some methods (Gradient, DeepLift, GradientShap, InputXGradient, Guided Backpropagation and LRP) seem to give a higher attribution in the pixels inside the ground-truth than to the rest of the pixels. For those methods, some lighter regions of the brain, such as the grey matter, also have higher attribution than the rest of the brain. Thus, we can suppose that the lesions are highlighted in these methods because their pixels have higher intensity in the input image, making them more salient than other brain regions. Saliency and Deconvolution have a more homogeneous attribution distribution and do not highlight the lesions.

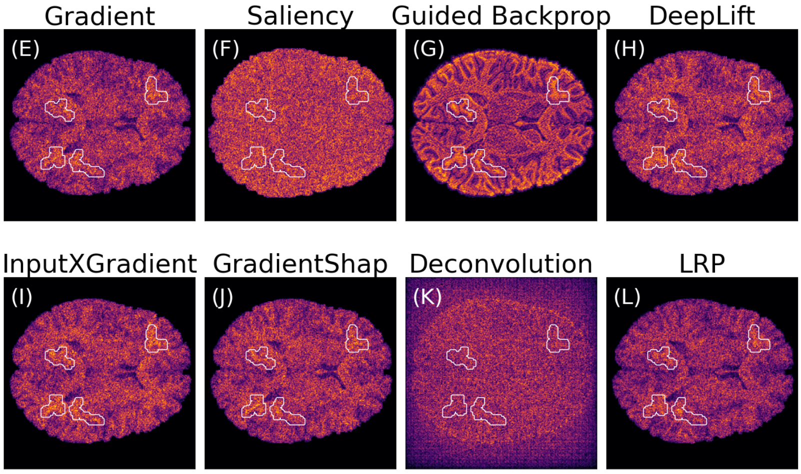

Figure S3. Baseline importance heat maps obtained from XAI methods applied to a randomly initialized, untrained model.

7 MODELS FINE-TUNED WITH SIMILAR PERFORMANCE

Figure S4 shows Pearson correlations between the average explanation accuracy of individual XAI methods and the test classification accuracy achieved by the underlying model for every combination of data used for pre-training and XAI methods. For almost all combinations, very strong positive correlations > 0.9 can be observed, representing a potential confound for the analysis of explanation performance as a function of the degree of model fine-tuning. Therefore, we control for unequal classification accuracy by selecting five models per fine-tuning degree that achieve similar test accuracy, close to 90%. The classification performance of these models is presented in Table S1. The models selected with this criterion for the lowest degree of fine-tuning (1conv) are unchanged, as these models do not reach the specified performance. The classification performance of the selected models for larger degrees of fine-tuning is between 88% and 89%.

Table S1. Average test classification accuracy of the three selected fine-tuned models that performed similarly and corresponding learning rates for several degrees of fine-tuning

		1conv	2conv	3conv	4conv	all
ImageNet	LR	0.020	0.008	0.008	0.008	0.004
	Test Acc	75.00%	89.10%	89.70%	89.00%	89.40%
		74.90%	88.10%	89.60%	89.10%	89.40%
		74.80%	88.80%	88.90%	88.20%	89.00%
	mean	74.90%	88.67%	89.40%	88.77%	89.27%
MRI	LR	0.038	0.030	0.015	0.030	0.010
	Test Acc	82.80%	90.50%	86.70%	88.20%	88.20%
		79.50%	89.60%	89.20%	87.40%	90.50%
		79.60%	89.20%	87.60%	88.80%	89.60%
	mean	80.63%	89.77%	87.83%	88.13%	89.43%

Figure S5 is analogous to Figure 3 but is based on models that achieved comparable classification accuracy across fine-tuning degrees. Compared to Figure 3, importance maps obtained for models that

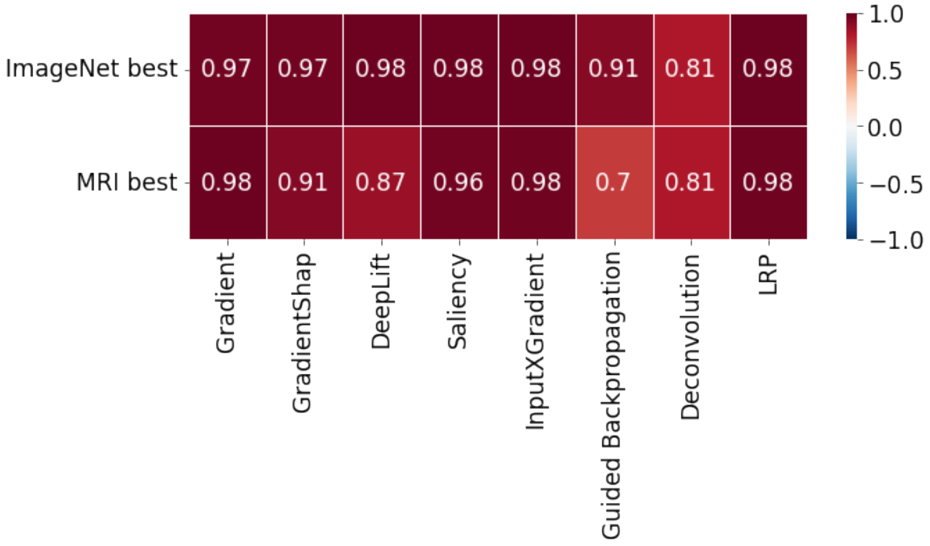

Figure S4. Pearson correlation between the mean value of the explanation performance for every XAI method and the average classification performance of the five models. The first row corresponds to the models with the best classification performance pre-trained with ImageNet data. The second row corresponds to the models with the best classification performance pre-trained with MRI data.

were pre-trained on ImageNet data appear to be degraded, while corresponding maps obtained for models that were pre-trained on MR images still closely resemble those obtained for best performing models as depicted in Figure 3.

Figure S6 shows corresponding quantitative results for models that achieved comparable performance across fine-tuning degrees. The attained explanation performance of models that were pre-trained with MR images is similar to the explanation performance obtained with the best performing models as depicted in Figure 4. The same is not true for models pre-trained with ImageNet data. Even though these models still achieve high classification accuracies of almost 90%, explanations derived from the resulting models seem to be much worse than for the best performing models (which achieve accuracies close to 100%). Importantly, for comparable classification accuracy, models pre-trained on ImageNet data were also consistently achieved lower explanation performance than models pre-trained on MR images. Overall, explanations obtained from models pre-trained with images from a similar domain may have a similar quality throughout a bigger range of classification performance values compared to explanations obtained from models pre-trained with images from different domains. In other words, this would indicate that using within-domain images to pre-train models can be beneficial with respect to the interpretability of a model even if the resulting models achieves lower classification performance.

8 SOCIETAL IMPACT

Our work mainly contributes to the evaluation of XAI methods, to avoid potential negative consequences of using such methods in high-stake decision environments, which we see as outweighing potential negative societal impact by a large margin. On the other hand, we propose one specific and narrow definition of feature importance and to that extent ‘define’ how XAI methods ‘should’ work. It is conceivable that this could lead to potential rejections of future ideas or approaches that might be able to generate superior explanations apart from the concept employed in this work.

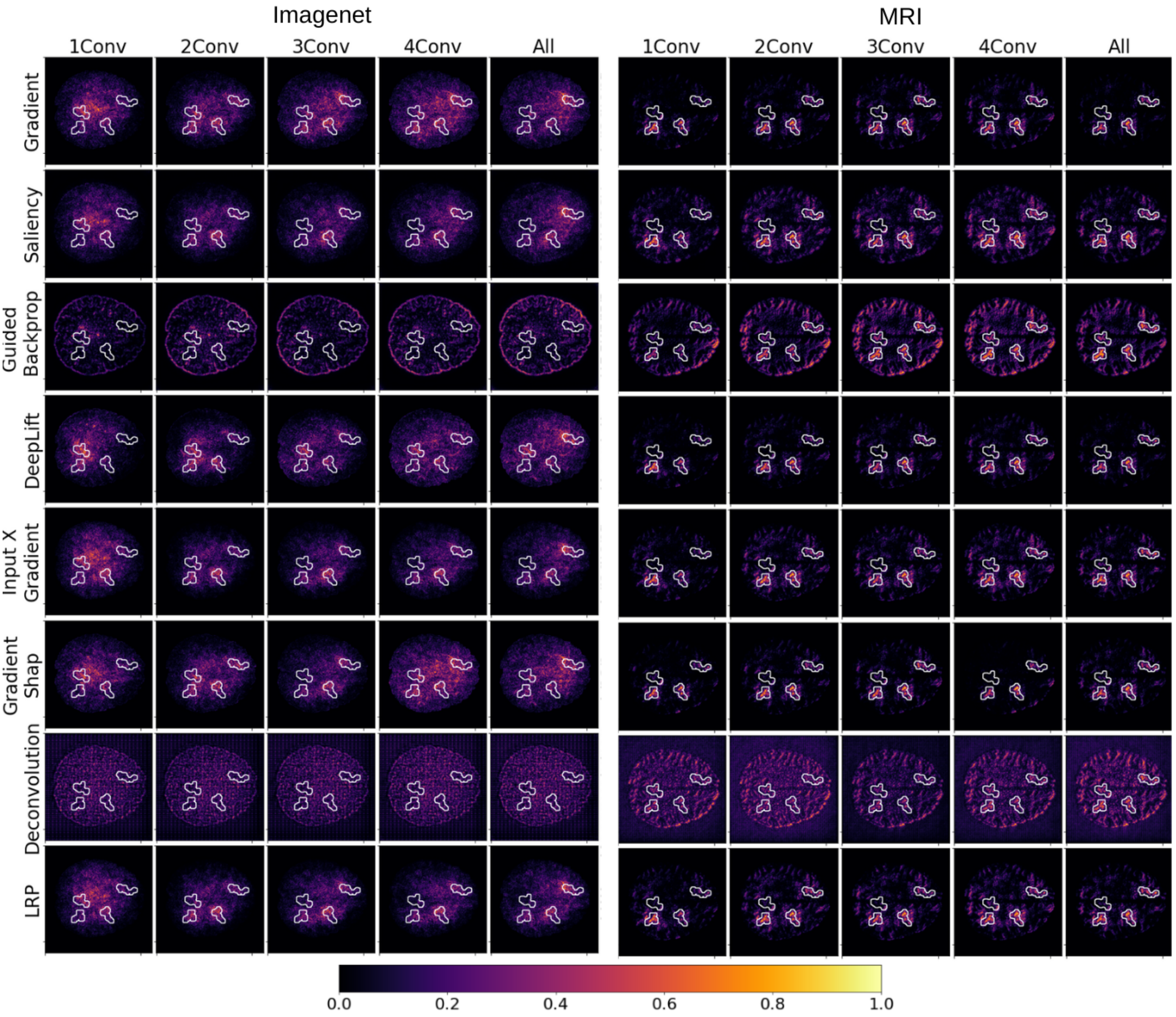

Figure S5. Examples of 'explanation' heat maps representing importance scores attributed to individual inputs by popular XAI methods for several degrees of fine-tuning of the VGG-16 architecture. Models using different degrees of fine-tuning were selected to achieve comparable test classification accuracies. Each row corresponds to an XAI method, whereas each column corresponds to a different degree of fine-tuning starting from fine-tuning 1 convolution block (1conv) and increasing to fine-tuning all layers (all). The image is divided into two vertical blocks, where importance maps obtained from models pre-trained with ImageNet data are depicted on the left side, and importance maps obtained from models pre-trained with MR images are depicted on the right side.

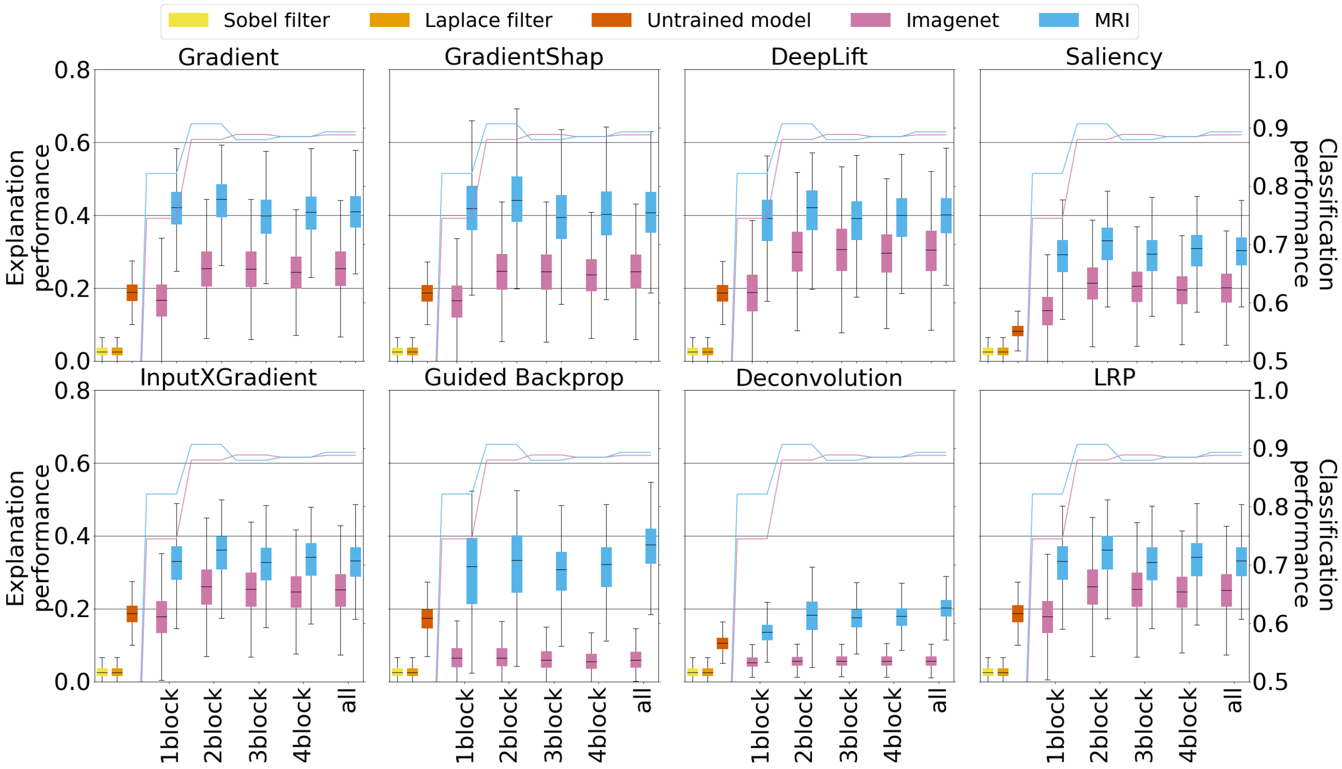

Figure S6. Quantitative explanation performance for various XAI methods applied to five models per degree of fine-tuning, which were selected to achieve comparable test classification accuracy. The blue line and boxplots correspond, respectively, to the classification performance (accuracy) and explanation performance (precision) derived from models pre-trained with MRI data, whereas the pink line and boxlots correspond analogously to classification and explanation performance for models pre-trained with ImageNet data. The other three boxplots correspond to the performance of baseline heat maps. Yellow and orange color correspond to the Sobel and Laplace filters respectively, and red color corresponds to the model with random weights.

9 THE ML PAPER REPRODUCIBILITY CHECKLIST (AS PER Pineau et al. (2021), V2.0)

1. For all models and algorithms presented, check if you include:
   a. A clear description of the mathematical setting, algorithm, and/or model. [Yes]
   b. A clear explanation of any assumptions. [Yes]
   c. An analysis of the complexity (time, space, sample size) of any algorithm [Yes]
2. For any theoretical claim, check if you include:
   a. A clear statement of the claim. [N/A]
   b. A complete proof of the claim. [N/A]
3. For all datasets used, check if you include:
   a. The relevant statistics, such as number of examples. [Yes]
   b. The details of train / validation / test splits. [Yes]
   c. An explanation of any data that were excluded, and all pre-processing step [Yes]
   d. A link to a downloadable version of the dataset or simulation environment [Yes]
   e. For new data collected, a complete description of the data collection process, such as instructions to annotators and methods for quality control [Yes]
4. For all shared code related to this work, check if you include:
   a. Specification of dependencies. [Yes]
   b. Training code. [Yes]
   c. Evaluation code. [Yes]
   d. (Pre-)trained model(s). [Yes]
   e. README file includes table of results accompanied by precise command to run to produce those results [Yes]
5. For all reported experimental results, check if you include:
   a. The range of hyper-parameters considered, method to select the best hyper-parameter configuration, and specification of all hyper-parameters used to generate results. [Yes]
   b. The exact number of training and evaluation runs [Yes]
   c. A clear definition of the specific measure or statistics used to report results. [Yes]
   d. A description of results with central tendency (e.g. mean) and variation (e.g. error bars). [Yes]
   e. The average runtime for each result, or estimated energy cost. [Yes]
   f. A description of the computing infrastructure used. [Yes]

10 DATASHEET (TEMPLATE BY Gebru et al. (2021))

Motivation

For what purpose was the dataset created? Was there a specific task in mind? Was there a specific gap that needed to be filled? Please provide a description.

With the rise of deep neural networks (DNNs) and their deployment in high-stake decision environments, many researchers and developers ask for techniques that shed light on the inner workings of such complex models, as they are commonly perceived as opaque 'black-box' models. Thus, XAI methods were developed to approach this problem. However, the lack of clarity on what formal problem the field of XAI is supposed to solve led to a branch of research to quantitatively validate such methods. Along this line of research, we propose a dataset where class-relevant features are known by construction and can serve as ground-truth explanations.

A common practice of applying ML models for the medical domain, here MRI, where labelled data are scarce, because of the cumbersome and expensive acquisition process, is, to deploy transfer learning techniques. The main purpose of the provided dataset is therefore to provide a validation framework, for novel XAI approaches aiming to explain decision made by pre-trained ML models that are fine-tuned to solve a specific MRI classification task.

Who created this dataset (e.g., which team, research group) and on behalf of which entity (e.g., company, institution, organization)?

This dataset has been constructed by the research group "Quality AI Labs" at the Technische Universität Berlin[1]. Furthermore, the 'base' dataset, the background images, was provided by the Human Connectome Project.

Who funded the creation of the dataset? If there is an associated grant, please provide the name of the grantor and the grant name and number.

Data were provided in part by the Human Connectome Project, WU-Minn Consortium (Principal Investigators: David Van Essen and Kamil Ugurbil; 1U54MH091657) funded by the 16 NIH Institutes and Centers that support the NIH Blueprint for Neuroscience Research; and by the McDonnell Center for Systems Neuroscience at Washington University.

This result is part of a project that has received funding from the European Research Council (ERC) under the European Union's Horizon 2020 research and innovation programme (Grant agreement No. 758985), the German Federal Ministry for Economy and Climate Action (BMWK) in the frame of the QI-Digital Initiative, and the Heidenhain Foundation.

Composition

What do the instances that comprise the dataset represent (e.g., documents, photos, people, countries)? Are there multiple types of instances (e.g., movies, users, and ratings; people and interactions between them; nodes and edges)? Please provide a description.

This dataset only contains brain images with artificial lesions. Ground-truth explanations come in the form of lesion maps represented as images as well.

How many instances are there in total (of each type, if appropriate)?

Our dataset consists of a training, validation and holdout dataset that encompasses 2500 images each. For the underlying HCP data, we chose 601 subjects for training, 206 for validation, and 201 for holdout. From these subjects, we generated 24924 slices for the training dataset, 8539 slices for the validation dataset, and 8319 slices for the holdout dataset. Where the holdout set and 2500 images from the validation set combined represent our provided lesion dataset. The training dataset consists of 24924 slices and is used for model pre-training and takes $14.5\,GB$, the validation dataset $4.98\,GB$ and the holdout set $4.85\,GB$.

Does the dataset contain all possible instances or is it a sample (not necessarily random) of instances from a larger set? If the dataset is a sample, then what is the larger

[1] https://www.tu.berlin/uniml

set? Is the sample representative of the larger set (e.g., geographic coverage)? If so, please describe how this representativeness was validated/verified. If it is not representative of the larger set, please describe why not (e.g., to cover a more diverse range of instances, because instances were withheld or unavailable).

For the creation of the dataset, we leveraged all subjects contained in the HCP data.

What data does each instance consist of? "Raw" data (e.g., unprocessed text or images) or features? In either case, please provide a description.

Each instance consists of a list `[image, gender, age]` where the image is a grayscale MRI slice, gender is a binary variable with 1 for male and 0 for female, and age is a binary variable as well, and takes the value 0 for the subjects age ≤ 27.5 and 1 otherwise.

Is there a label or target associated with each instance? If so, please provide a description.

We assign labels according to the shape of the artificial lesions incorporated into the brain images. For images comprising round lesions we assign the label 0, and for images comprising irregular lesions we assign the label 1.

Is any information missing from individual instances? If so, please provide a description, explaining why this information is missing (e.g., because it was unavailable). This does not include intentionally removed information, but might include, e.g., redacted text.

No.

Are relationships between individual instances made explicit (e.g., users' movie ratings, social network links)? If so, please describe how these relationships are made explicit.

The Open Access HCP dataset does not contain any relational information.

Are there recommended data splits (e.g., training, development/validation, testing)? If so, please provide a description of these splits, explaining the rationale behind them.

We already provide a standard data split: training/validation and holdout (testing) set.

Are there any errors, sources of noise, or redundancies in the dataset? If so, please provide a description.

The HCP dataset consists of real-world MRI slices that are naturally suffering from recording artefacts and potential pre-processing perturbations.

Is the dataset self-contained, or does it link to or otherwise rely on external resources (e.g., websites, tweets, other datasets)? If it links to or relies on external resources, a) are there guarantees that they will exist, and remain constant, over time; b) are there official archival versions of the complete dataset (i.e., including the external resources as they existed at the time the dataset was created); c) are there any restrictions (e.g., licenses, fees) associated with any of the external resources that might apply to a future user? Please provide descriptions of all external resources and any restrictions associated with them, as well as links or other access points, as appropriate.

Newer versions of this dataset, which are not planned so far, will rely on the HCP. Apart from that, our dataset is self-contained.

Does the dataset contain data that might be considered confidential (e.g., data that is protected by legal privilege or by doctor-patient confidentiality, data that includes the content of individuals non-public communications)? If so, please provide a description.

Our dataset contains brain slices of individuals, though names and other meta-data that can identify individuals are not contained, also the MRI records are de-faced and considered de-identified under HIPAA Elam et al. (2021).

Does the dataset contain data that, if viewed directly, might be offensive, insulting, threatening, or might otherwise cause anxiety? If so, please describe why.

No.

Does the dataset relate to people? If not, you may skip the remaining questions in this section.

Yes.

Does the dataset identify any subpopulations (e.g., by age, gender)? If so, please describe how these subpopulations

are identified and provide a description of their respective distributions within the dataset.

The provided training dataset consists of $57\,\%$ slices of male brain images, the validation dataset consists of $55\,\%$ slices of male brain images, and the holdout dataset consists of $48\,\%$ slices of male brain images.

Is it possible to identify individuals (i.e., one or more natural persons), either directly or indirectly (i.e., in combination with other data) from the dataset? If so, please describe how.

Even though the dataset only consists of brain image slices and binary sex and age variables, it is possible to identify individuals.

Does the dataset contain data that might be considered sensitive in any way (e.g., data that reveals racial or ethnic origins, sexual orientations, religious beliefs, political opinions or union memberships, or locations; financial or health data; biometric or genetic data; forms of government identification, such as social security numbers; criminal history)? If so, please provide a description.

No.

Collection Process

How was the data associated with each instance acquired? Was the data directly observable (e.g., raw text, movie ratings), reported by subjects (e.g., survey responses), or indirectly inferred/derived from other data (e.g., part-of-speech tags, model-based guesses for age or language)? If data was reported by subjects or indirectly inferred/derived from other data, was the data validated/verified? If so, please describe how.

The brain slices or images, were extracted from MRI recordings acquired via MRI devices by the HCP project (for more details see Van Essen et al. (2013)). Otherwise, the lesions were artificially generated (see section **??** of the main text).

What mechanisms or procedures were used to collect the data (e.g., hardware apparatus or sensor, manual human curation, software program, software API)? How were these mechanisms or procedures validated?

We created lesions algorithmically. For the details of the recording mechanisms of the MRI recordings, consult the corresponding publication of the HCP project Van Essen et al. (2013).

If the dataset is a sample from a larger set, what was the sampling strategy (e.g., deterministic, probabilistic with specific sampling probabilities)?

The brain slice images were randomly sampled from a larger dataset. The corresponding seeds are made available via our Github repository: `https://github.com/Marta54/Pretrain_XAI_gt`.

Who was involved in the data collection process (e.g., students, crowdworkers, contractors) and how were they compensated (e.g., how much were crowdworkers paid)?

No data were collected from our lab members, but for the HCP project, it is not apparent who was involved in the data collection process (see Van Essen et al. (2013)).

Over what timeframe was the data collected? Does this timeframe match the creation timeframe of the data associated with the instances (e.g., recent crawl of old news articles)? If not, please describe the timeframe in which the data associated with the instances was created.

A timeline for the HCP project is provided by Elam et al. (2021).

Were any ethical review processes conducted (e.g., by an institutional review board)? If so, please provide a description of these review processes, including the outcomes, as well as a link or other access point to any supporting documentation.

Yes, see Van Essen et al. (2013); Elam et al. (2021). However, here we only use de-identified data with respect to HIPAA.

Does the dataset relate to people? If not, you may skip the remaining questions in this section.

Yes.

Did you collect the data from the individuals in question directly, or obtain it via third parties or other sources (e.g., websites)?

The HCP project Van Essen et al. (2013) is a third-party project that provides access to high-quality brain imaging data, which we based our dataset on.

Were the individuals in question notified about the data collection? If so, please describe (or show with screenshots or other information) how notice was provided, and provide a link or other access point to, or otherwise reproduce, the exact language of the notification itself.

Participants explicitly expressed consent to participate in the HCP project Van Essen et al. (2013).

Did the individuals in question consent to the collection and use of their data? If so, please describe (or show with screenshots or other information) how consent was requested and provided, and provide a link or other access point to, or otherwise reproduce, the exact language to which the individuals consented.

For the HCP data, the informed consent document explicitly states that the data of the participants will be shared publicly over the internet Elam et al. (2021).

If consent was obtained, were the consenting individuals provided with a mechanism to revoke their consent in the future or for certain uses? If so, please provide a description, as well as a link or other access point to the mechanism (if appropriate).

Individuals provided informed consent with the knowledge that their data will be distributed via the internet. As the data use terms Van Essen et al. (2013) allows for the re-distribution of derivative work under the same license, even if a participant withdraws their informed consent, deleting their data completely is potentially infeasible.

Has an analysis of the potential impact of the dataset and its use on data subjects (e.g., a data protection impact analysis) been conducted? If so, please provide a description of this analysis, including the outcomes, as well as a link or other access point to any supporting documentation.

For the HCP data, privacy concerns were discussed leading to two tiers of data access: ”Open Access Data” and ”Restricted Access Data” Elam et al. (2021). We use the Open Access dataset.

Preprocessing/cleaning/labeling

Was any preprocessing/cleaning/labeling of the data done (e.g., discretization or bucketing, tokenization, part-of-speech tagging, SIFT feature extraction, removal of instances, processing of missing values)? If so, please provide a description. If not, you may skip the remainder of the questions in this section.

Yes. From the initial HCP data, we selected only slices with less than 55% black pixels. From these, their intensity was changed so that every slice had a similar average pixel intensity. These slices were also reshaped from 310×260 pixels to 270×270. The HCP images used were also previously pre-processed with FreeSurfer 5.1 software and registered to MNI152 space using FSL's linear FLIRT tool, followed by FNIRT algorithm as described in Van Essen et al. (2013). The HCP data was also defaced using the technique described in Milchenko and Marcus (2013).

Was the "raw" data saved in addition to the preprocessed/cleaned/labeled data (e.g., to support unanticipated future uses)? If so, please provide a link or other access point to the "raw" data.

We saved the image slices selected from the HCP dataset that we used. These images are available on our provided OSF repository [2] and are contained in the folder named Pre-training dataset.

The raw data of our dataset consists of the slices, gender and age corresponding to those slices and the slices were used both to pre-train the model and to serve as a background to our synthetic lesions. Note that the slices used to pre-train a model to the task of classifying female and male slices were not the same slices used to fine-tune the model, as we only used the holdout set and parts of the validation set.

Is the software used to preprocess/clean/label the instances available? If so, please provide a link or other access point.

Yes. The pre-processing of our dataset can be found in `https://github.com/Marta54/`

[2] https://www.doi.org/10.17605/OSF.IO/XNWAJ

Pretrain_XAI_gt, while the pre-process made to the HCP data can be followed from the HCP paper Van Essen et al. (2013).

Uses

Has the dataset been used for any tasks already? If so, please provide a description.

Our dataset has not been used for other tasks yet. The HCP dataset is a widely used research dataset Elam et al. (2021).

Is there a repository that links to any or all papers or systems that use the dataset? If so, please provide a link or other access point.

An aggregated overview of published papers using the HCP dataset can be found in Elam et al. (2021). The HCP website itself provides a list of selected publications using the HCP dataset: https://www.humanconnectome.org/study/hcp-young-adult/publications

What (other) tasks could the dataset be used for?

The data we provide is dedicated to analyzing XAI methods applied to pre-trained models that are fine-tuned on our dataset. Technically, one could employ this dataset to perform benchmarks for model fine-tuning on the MRI domain.

Is there anything about the composition of the dataset or the way it was collected and preprocessed/cleaned/labeled that might impact future uses? For example, is there anything that a future user might need to know to avoid uses that could result in unfair treatment of individuals or groups (e.g., stereotyping, quality of service issues) or other undesirable harms (e.g., financial harms, legal risks) If so, please provide a description. Is there anything a future user could do to mitigate these undesirable harms?

For our provided pre-training dataset we were aiming to balance the sampled brain slides according to the subjects' sex. For reasoning of the composition of the HCP dataset we refer to Van Essen et al. (2013).

Are there tasks for which the dataset should not be used? If so, please provide a description.

Yes, according to the data access terms of the HCP project, it is prohibited to try to establish the identity of the included human subjects (e.g. see Van Essen et al. (2013)).

Distribution

Will the dataset be distributed to third parties outside of the entity (e.g., company, institution, organization) on behalf of which the dataset was created? If so, please provide a description.

Yes. We aim to make our dataset publically available under an open-access license complying with HCP data use terms[3].

How will the dataset will be distributed (e.g., tarball on website, API, GitHub) Does the dataset have a digital object identifier (DOI)?

We plan to host a general benchmark suite for XAI methods to evaluate the quality of produced explanations. In the realm of that benchmark suite, we aim to offer our MRI dataset as well, which will be hosted at the Physikalisch-Technische Bundesanstalt in Germany.

When will the dataset be distributed?

Currently, the MRI dataset is only available for the reviewers but we plan to provide a publically accessible dataset, preferably hosted via the Physikalisch-Technische Bundesanstalt in Germany, made available by publication of this manuscript.

Will the dataset be distributed under a copyright or other intellectual property (IP) license, and/or under applicable terms of use (ToU)? If so, please describe this license and/or ToU, and provide a link or other access point to, or otherwise reproduce, any relevant licensing terms or ToU, as well as any fees associated with these restrictions.

We would like to publish our dataset under a Creative Commons license.

[3] https://www.humanconnectome.org/study/hcp-young-adult/data-use-terms

Have any third parties imposed IP-based or other restrictions on the data associated with the instances? If so, please describe these restrictions, and provide a link or other access point to, or otherwise reproduce, any relevant licensing terms, as well as any fees associated with these restrictions.

The HCP dataset requires the agreement of the license provided by the HCP project[4].

Do any export controls or other regulatory restrictions apply to the dataset or to individual instances? If so, please describe these restrictions, and provide a link or other access point to, or otherwise reproduce, any supporting documentation.

The Open Access HCP dataset is de-identified and the project's data access terms apply.

Maintenance

Who will be supporting/hosting/maintaining the dataset?

The dataset is supported by the authors and by the QAI Labs research group. Currently, the dataset is hosted on OSF for the review process. It is planned to host the dataset on servers of the German governance institute the "Physikalisch-Technische Bundesanstalt", to ensure long-time availability. If reviewers have concrete concerns about this hosting strategy, the usage of the "Open Science Framework" (OSF[5]) can be an alternative hosting service.

How can the owner/curator/manager of the dataset be contacted (e.g., email address)?

The authors of this dataset can be reached at the e-mail address: `haufe@tu-berlin.de`.

Is there an erratum? If so, please provide a link or other access point.

If errors are found an erratum will be added to the website containing all meta-information about this dataset.

Will the dataset be updated (e.g., to correct labeling errors, add new instances, delete instances)? If so, please describe how often, by whom, and how updates will be communicated to users (e.g., mailing list, GitHub)?

As we plan the dataset as part of an XAI benchmark suite hosted at the Physikalisch-Technische Bundesanstalt in Germany, updates and error corrections will be part of the maintenance of this benchmark platform, i.e. a changelog can be provided via the corresponding website.

If the dataset relates to people, are there applicable limits on the retention of the data associated with the instances (e.g., were individuals in question told that their data would be retained for a fixed period of time and then deleted)? If so, please describe these limits and explain how they will be enforced.

Participants of the HCP project had to sign an informed consent form, which explicitly states that their data will be made public via the Internet, thus its access is not constrained by any limits other than the data use terms[6]

Will older versions of the dataset continue to be supported/hosted/maintained? If so, please describe how. If not, please describe how its obsolescence will be communicated to users.

Older versions of this dataset could be hosted via the website of the aforementioned XAI benchmark.

If others want to extend/augment/build on/contribute to the dataset, is there a mechanism for them to do so? If so, please provide a description. Will these contributions be validated/verified? If so, please describe how. If not, why not? Is there a process for communicating/distributing these contributions to other users? If so, please provide a description.

As mentioned above, we aim to publish this dataset under a Creative Commons license, which yields an opportunity for other researchers to freely access this dataset and create derivative work.

[4] `https://www.humanconnectome.org/study/hcp-young-adult/document/wu-minn-hcp-consortium-open-access-data-use-terms`

[5] `osf.io`

[6] `https://www.humanconnectome.org/study/hcp-young-adult/document/wu-minn-hcp-consortium-open-access-data-use-terms.`

REFERENCES

Elam, J. S., Glasser, M. F., Harms, M. P., Sotiropoulos, S. N., Andersson, J. L., Burgess, G. C., et al. (2021). The human connectome project: A retrospective. *NeuroImage* 244, 118543

Gebru, T., Morgenstern, J., Vecchione, B., Vaughan, J. W., Wallach, H., Iii, H. D., et al. (2021). Datasheets for datasets. *Communications of the ACM* 64, 86–92

Milchenko, M. and Marcus, D. (2013). Obscuring surface anatomy in volumetric imaging data. *Neuroinformatics* 11, 65–75. doi:10.1007/s12021-012-9160-3

Pineau, J., Vincent-Lamarre, P., Sinha, K., Larivière, V., Beygelzimer, A., d'Alché Buc, F., et al. (2021). Improving reproducibility in machine learning research (a report from the neurips 2019 reproducibility program). *The Journal of Machine Learning Research* 22, 7459–7478

Van Essen, D. C., Smith, S. M., Barch, D. M., Behrens, T. E., Yacoub, E., and Ugurbil, K. (2013). The WU-Minn human connectome project: An overview. *NeuroImage* 80, 62–79. doi:10.1016/j.neuroimage.2013.05.041
